# Angiogenic inflammation and formation of necrosis in the tumor microenvironment influence patient survival after radical surgery for de novo hepatocellular carcinoma in non-cirrhosis

**DOI:** 10.1186/s12957-019-1756-8

**Published:** 2019-12-12

**Authors:** Georgi Atanasov, Karoline Dino, Katrin Schierle, Corinna Dietel, Gabriela Aust, Johann Pratschke, Daniel Seehofer, Moritz Schmelzle, Hans-Michael Hau

**Affiliations:** 1 Department of Visceral, Transplantation, Thoracic and Vascular Surgery, University Hospital Leipzig, Leipzig, Germany; 2 Department of Surgery, Campus Charité Mitte und Campus Virchow-Klinikum, Charité – Universitätsmedizin Berlin, Charitéplatz 1, 10117 Berlin, Germany; 3 Berlin Institute of Health, Berlin, Germany; 4 Institute of Pathology, University Hospital Leipzig, Leipzig, Germany; 5 Department of Surgery, Research Laboratories, University of Leipzig, Leipzig, Germany

**Keywords:** Hepatocellular carcinoma, Tumor-infiltrating macrophages, TIE2-expressing monocytes, Tumor necrosis, Angiopoietins, Angiogenesis, Prognosis

## Abstract

**Background:**

Tumor escape mechanisms mediated in the tumor microenvironment can significantly reduce the capacity of the anti-tumor function of the immune system. TIE2-expressing monocytes (TEMs), related angiopoietins, and tumor necrosis are considered to have a key role in this process. We aimed to investigate the abundance and clinical significance of these biomarkers in hepatocellular carcinoma (HCC).

**Methods:**

In this retrospective study, 58 HCC patients received surgery with a curative intent. The abundance of TEMs, angiopoietin-1 and -2 were detected in tumor specimens of the HCC patients (*n* = 58), and together with the occurrence of histologic tumor necrosis, were associated with established clinicopathological characteristics and survival.

**Results:**

Patients with HCC characterized by necrosis and TEMs revealed reduced both overall survival and recurrence-free survival (all *p* < 0.05). Angiopoietins and TEMs were associated with metastatic and recurrent HCC. Furthermore, the formation of histologic tumor necrosis was associated with advanced tumor stage and density of TEMs (all *p* < 0.05).

**Conclusions:**

Histologic tumor necrosis, TEMs, and related angiopoietins were associated with multiple HCC parameters and patient survival. The tumor necrosis–TEM–angiopoietin axis may offer a novel diagnostic modality to predict patient outcome after surgery for HCC.

## Background

Liver cirrhosis is an established risk factor for HCC. However, HCC also arises de novo in non-cirrhotic livers in approximately 20% of all cases, with host inflammatory responses having a key importance in hepatocarcinogenesis [1–3]. There is a rising clinical interest in patients with de novo HCC, because this subgroup commonly presents at an advanced stage, as surveillance is usually not performed in patients without liver disease. Tumor growth in these non-cirrhotic patients is clinically silent in its early stages because of the lack of symptoms and compensated hepatic function. On the other hand, the importance of non-alcoholic steatohepatitis (NASH) in driving the process of hepatocarcinogenesis has been recently recognized and put into a causal context with de novo HCC [4]. Of note, NASH-driven hepatocarcinogenesis is mechanistically involved in the process of necrosis formation in the tumor microenvironment, and the latter has also been related to enhanced infiltration with immune-competent cells [5–9]. Furthermore, experimental studies reported on novel angiogenic pathways playing a key role in de novo or NASH-driven hepatocarcinogenesis, implicating the complex immunologic mechanisms involved in cancer progression [10–13].

The significance of complex angiogenic properties of the tumor microenvironment in HCC has come to the fore in recent years. Tumor angiogenesis has been validated as an attractive therapeutic target in the process of hepatocarcinogenesis, mainly in clinical trials targeting the vascular endothelial growth factor (VEGF) pathway [14]. However, a deeper insight into the biology of solid cancer reveals that the host cellular immune competence in the tumor microenvironment is mechanistically intertwined with angiogenesis and necrosis formation, and the blockade of only one functional pathway does not reach the desired long-term efficacy in cancer patients. Recently, the angiopoietin family of ligands, angiopoietin-1 and -2, has been demonstrated to selectively activate the endothelial cell membrane receptor tyrosine kinase TIE2 and to espouse tumor progression [15, 16]. In the scope of tumor angiogenesis and metastasis, this angiopoietin axis-TIE growth factor receptor pathway represents the key regulator of pathological vascular permeability and remodeling, and its pharmacological blockade is in clinical development in oncologic settings [14]. In this scenario, the role of novel angiomodulatory monocytes/macrophages subsets in hepatocarcinogenesis is vastly unknown. Angiogenic immune–competent cells represent a unique subpopulation of tumor-infiltrating bone marrow-derived myeloid cells, which differ from the classical tumor-associated macrophages (TAMs) [17]. These cellular effectors have immense angiogenic potential, express functionally active TIE2-expressing macrophages (TEMs) and directly respond to angiopoietin activity [18]. Interestingly, the tissue-infiltrating fraction of TEMs, which promotes angiogenesis and carcinogenesis, is localized only in the tumor microenvironment and not in healthy tissues [17, 18]. In line with this, our data demonstrated significant influence of TEMs on patient outcome in other cancer types [19–21]. Thus, the study of TEMs in HCC may identify attractive targets for immunologic checkpoint inhibition.

In rapidly growing tumors, the formation of necrosis can be attributed to the relative hypoperfusion in the vicinity of the tumor. However, novel scientific results demonstrate that the nature of necrosis formation in the vicinity of the tumor is much more complex, as its occurrence is functionally mediated by infiltrating monocytes/macrophages and delineated its role as a prognosticator of aggressiveness in primary solid tumors [22–24]. The presence of tumor necrosis in HCC may adequately characterize the tumor biology and provide additional beneficial prognostic information. Furthermore, we previously demonstrated the importance of histologic tumor necrosis and its relation to monocytes/macrophages in poorly vascularized human cholangiocarcinoma [25, 26]. However, HCC is in most cases a highly vascularized tumor [27]. Therefore, formation of necrosis might also be a typical feature of subsets of HCC. However, early stage and recurrence of HCC are difficult to detect by non-invasive imaging, and alpha-fetoprotein (AFP) as a surveillance biomarker has been removed from present guidelines because of its low sensitivity and specificity [28]. Novel biomarkers for the management and prognosis of HCC patients are needed, and thus, in the current work, we focused on the potential of tumor necrosis to be facilitated by immunological components of the tumor microenvironment (i.e., invading monocytes/macrophages).

Patients with de novo HCC are at enhanced risk for adverse disease outcome. Intriguingly, despite tumor necrosis induced by impaired tissue oxygen delivery, which is generally due to a compromised blood supply to cancerous tissue, recent studies also revealed that histologic tumor necrosis was associated with high levels of angiogenesis and increased inflammation in the tumor microenvironment [29, 30]. Interestingly, a strong association between liver steatosis and formation of tumor necrosis in de novo HCC in non-cirrhotic livers has been recently revealed [5]. Thus, possible immunologic coherence comprising tumor-infiltrating angiogenic monocytes/macrophages, associated molecular factors of angiogenesis and formation and extent of tumor necrosis could effectively characterize HCC patients. Recently, our group reported on the clinicopathologic characteristics of patients with de novo HCC and the clinical significance of tumor-infiltrating classical monocytes and lymphocytes in this hepatic pathology [31]. Insofar, the present work aimed to investigate the angiopoietin axis, related angiogenic TEMs, and tumor necrosis in de novo HCC in non-cirrhotic patients, and thus, deliver a deeper insight concerning the importance of novel immunologic biomarkers, which may help improve the personal risk and prognosis stratifications by defining subgroups of patients with beneficial or deleterious tumor characteristics.

## Methods

### Patients and tumor samples

The Institutional Ethics Committee approved the conduction of this study (no. 234-14-14072014). Patients (*n* = 58) with histopathologically confirmed HCC, who received a major hepatectomy with a curative intent, were included in our retrospective study (Additional file 3). HCC recurrence was diagnosed by a triphasic computed tomography (CT) scan and/or a magnetic resonance imaging (MRI) with a liver-specific contrast medium. We also routinely performed AFP serology. However, hepatocarcinogenesis is, in many cases, AFP silent [32]. Therefore, in our work, AFP negativity was not considered a reliable tool to exclude recurrent disease. In addition, in case of suspected tumor recurrence, CT and MRI were routinely conducted. None of the HCC patients in our study had liver cirrhosis, history of viral hepatitis, or was subjected to neoadjuvant radio- and/or chemotherapy before tumor resection. The complete inclusion and exclusion criteria of our study are described in the Additional file 3. Paraffin-embedded tissue blocks containing a representative HCC sample were retrieved from the archives of the Institute of Pathology. Histological assessment of frequencies of cellular infiltrates and angiopoietins and the presence of tumor necrosis in the HCC specimens was carried out by two investigators (KD and GA), with training in histopathology, and an independent pathologist (KS), without knowledge of the patient outcome or the corresponding clinicopathological characteristics.

### Immunohistology and quantification of TIE2-expressing monocytes and angiopoietins

Protocols for immunohistology and density quantification of cellular infiltrates were carried out as described [6–8]. Briefly, tissue TEMs were double immunostained for CD14 and TIE2, and antibodies for angiopoietin-1 and -2 were used to evaluate their abundance. The tumor-infiltrating front (TIF) and tumor central area (TCA) of the HCC specimens were analyzed separately. Cellular infiltrates and angiopoietin-positive staining were referred to as negative/absent in up to 5% positive cells (0–5% positive cells, score 0) and positive/present (>  5% positive cells, score 1). Next, patients were assigned to two different groups (negative or positive for TEMs, and angiopoietin-1- or -2-positive tumor cells). The Additional file 3 provides detailed information on antibodies, chemicals, reagents, and the process of histological evaluation in the current work.

### Occurrence of tumor necrosis

The presence of histological tumor necrosis in the studied HCC specimens was classified into two categories: 1, negative or 2, positive, as previously described [25, 26]. Patients were categorized into two groups (necrosis^+^ and necrosis^−^ groups) according to their “positive” or “negative” necrosis scores. In addition, the presence of liver steatosis was categorized into four degrees: absence of steatosis (0–5%), mild steatosis (>  5% and < 30%), moderate steatosis (30–60%), and severe steatosis (> 60%) [33], according to established histopathologic criteria [33].

### Statistical analysis

The IBM SPSS Statistics (Version 25/Year 2017) software program was used to perform the univariate and Kaplan–Meier survival analyses. The chi-squared (*χ*^2^) test was applied to determine whether there was a significant difference between the observed frequencies in the categorical variables (clinicopathologic characteristics) in our study. The Fischer test was applied when the number of patients in the subgroups was less than five (*n* < 5) in more than 25% of cases. Survival data were analyzed using the log-rank test. To investigate whether the studied variables and biomarkers qualify as independent prognostic factors, we performed a multivariate analysis in a step-wise manner. Parameters that significantly affected survival rates or showed a strong trend for significance in the univariate analysis were entered into a step-wise Cox regression hazard model. A difference was considered significant for *p* ≤ 0.05.

## Results

### TIE2-expressing monocytes and angiopoietins are associated with advanced histologic grading and tumor recurrence in patients with HCC

**Fig. 1:**
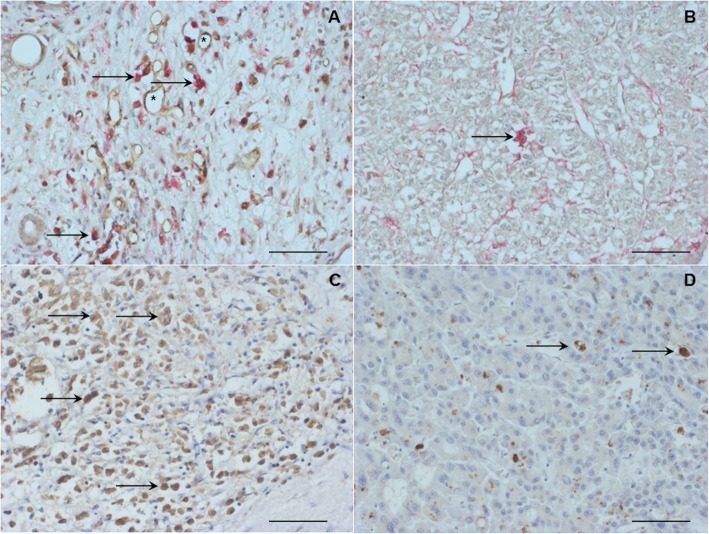
Immunohistological detection of infiltrating TIE2-expressing monocytes (TEMs), angiopoietins (left column: high density; right column: low density) in the tumor central area (TCA) of hepatocellular carcinoma (HCC) specimens. Arrows indicate positive staining and asterisks indicate microvessels. Scale bar, 50 μm. Therefore, the corresponding monocytes (i.e., TEMs) are visualized as cells that are double positive for CD14 and TIE2. This positivity in the immunohistology is seen as simultaneous red and brown staining (arrows). On the other hand, microvessels (i.e., endothelial cells) are positive only for TIE2 and are, thus, visualized as brown reactivity only (asterisks). **a** High density of TIE2^+^ monocytes in the TCA. HCC revealed a homogenous infiltration pattern of these cells with preference for the perivascular areas. **b** Low density of TIE2^+^ monocytes (arrows). **c** High angiopoietin-1 expression. **d** Low angiopoietin-1 expression

**Fig. 2:**
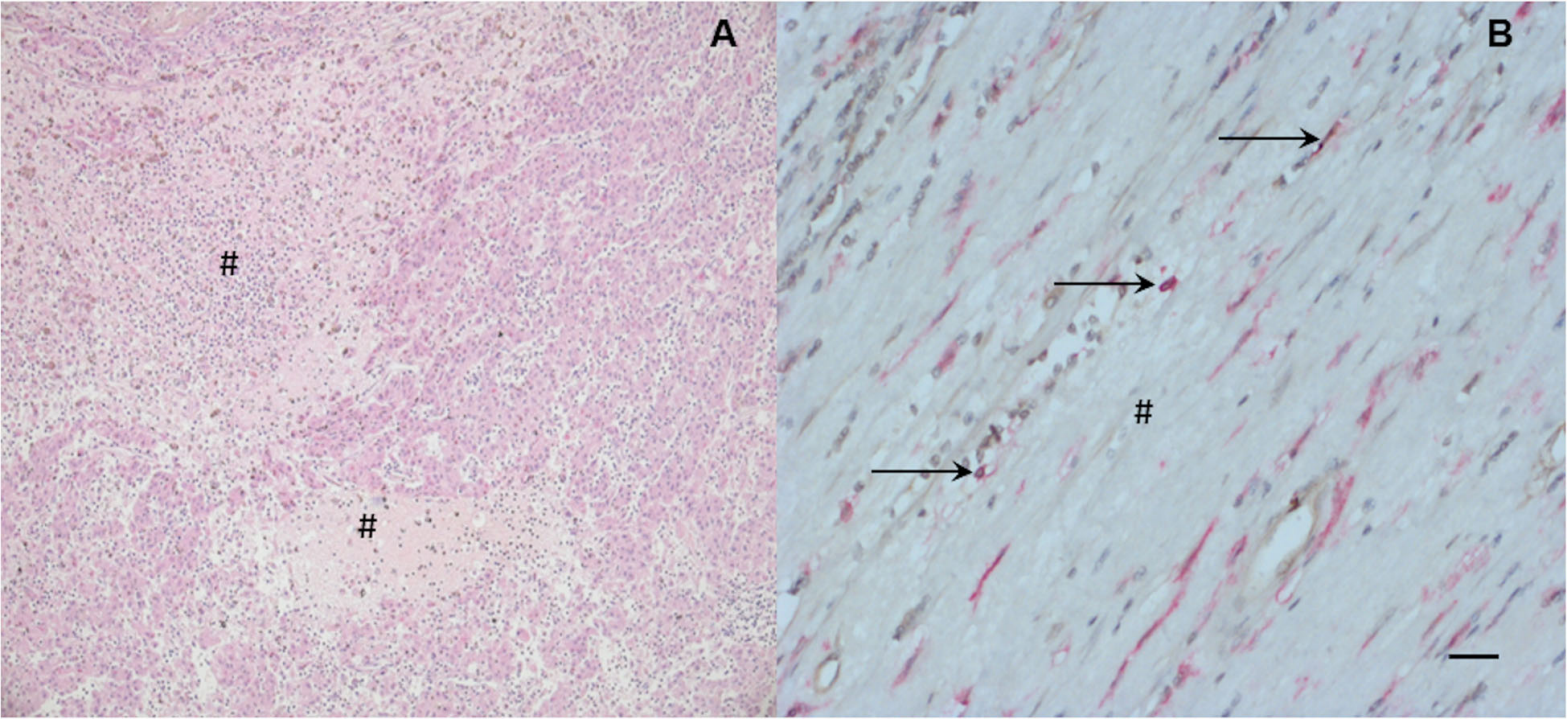
Detection of histologic tumor necrosis and infiltrating TIE2-expressing monocytes (TEMs) in hepatocellular carcinoma (HCC) specimens. Arrows indicate positive staining for TEMs (simultaneous red and brown staining) and diamond signs indicate tumor necrosis. Scale bar, 50 μm. **a** Tumor specimen with histological tumor necrosis. **b** Tumor specimen with histological tumor necrosis (diamond signs) and presence of TEMs (arrows)

Typical images for monocytes/macrophages and angiopoietins in HCC are shown in Figs. 1 and 2. The statistical data of the patients is summarized in Tables 1, 2, 3, and 4 and in the Additional file 3. In our study, the presence of TEMs was associated with enhanced incidences of both local tumor recurrence and overall tumor recurrence and worse histologic differentiation of HCC (Fig. 1a, b; Table 1). In the TEM^−^ group, 4/19 (21.1%) patients had an overall tumor recurrence, and in the TEM^+^ group, 19/39 (48.7%) patients exhibited this feature (*p* = 0.043). Moreover, the presence of TEMs was also associated with an unfavorable histologic differentiation of the tumor. In the TEM^+^ group, 35/39 (89.7%) patients were diagnosed with an advanced histologic grading, whereas in the TEM^−^ group, 12/19 (63.2%) patients had this feature (*p* = 0.015). In the current work, the presence of angiopoietin-1 in the TIF was associated with an enhanced incidence of local tumor recurrence (*p* = 0.007) (Fig. 1c, d; Table 2). After liver resection for HCC, in the ANG1^high^ group, 6/9 (66.7%) patients experienced local tumor recurrence, whereas in the ANG1^low^ group, only 11/49 (22.4%) patients had this phenomenon.

**Table 1:** Association of the presence of TIE2-expressing monocytes (TEMs) at the tumor-infiltrating front (TIF) with clinicopathological characteristics of patients with hepatocellular carcinoma (HCC) as determined by the chi-squared (*χ*^2^) test

Variable	TEM^+^/TIF	TEM^−^/TIF	*p*
No. of patients	39	19	
Patient- and tumor-related variables			
Patient age, years			0.094
≤ 60	17 (43.6%)	4 (21.1%)	
> 60	22 (56.4%)	15 (78.9%)	
Gender			0.029
Female	27 (69.2%)	18 (94.7%)	
Male	12 (30.8%)	1 (5.3%)	
Multiple tumor nodules			0.862
Positive	9 (23.1%)	4 (21.1%)	
Negative	30 (76.9%)	15 (78.9%)	
Tumor size, mm			0.319
≤ 50	6 (15.4%)	5 (26.3%)	
> 50	33 (84.6%)	14 (73.7%)	
Angioinvasion			0.113
Positive	23 (59.0%)	7 (36.8%)	
Negative	16 (41.0%)	12 (63.2%)	
Lymphangiosis carcinomatosa			0.727
Positive	12 (30.8%)	5 (26.3%)	
Negative	27 (69.2%)	14 (73.7%)	
Histologic differentiation			0.015
Well	4 (10.3%)	7 (36.8%)	
Moderate/poor	35 (89.7%)	12 (63.2%)	
Pathologic T stage			0.631
T1/T2	19 (48.7%)	10 (55.6%)	
T3/T4	20 (51.3%)	8 (44.4%)	
Pathologic N stage			0.315
Positive	2 (5.1%)	0 (0.0%)	
Negative	37 (94.9%)	19 (100.0%)	
Operative variables			
R status			0.464
Positive	7 (17.9%)	2 (10.5%)	
Negative	32 (82.1%)	17 (89.5%)	
Variables of patient outcome			
Local tumor recurrence			0.005
Positive	16 (41.0%)	1 (5.3%)	
Negative	23 (59.0%)	18 (94.7%)	
Overall tumor recurrence			0.043
Positive	19 (48.7%)	4 (21.1%)	
Negative	20 (51.3%)	15 (78.9%)	
Metastases			0.667
Positive	8 (20.5%)	3 (15.8%)	
Negative	31 (79.5%)	16 (84.2%)	

The Fischer test was applied when the number of patients in the subgroups was less than five (*n* < 5) in more than 25% of cases

**Table 2:** Association of relative angiopoietin-1 (ANG1) expression at the tumor-infiltrating front (TIF) or central area (TCA) with clinicopathological characteristics of the patients with hepatocellular carcinoma (HCC) as determined by the chi-squared (*χ*^2^) test

Variable	ANG1 high/TIF	ANG1 low/TIF	*p*
No. of patients	9	49	
Patient- and tumor-related variables			
Patient age, years			0.342
≤ 60	2 (22.7%)	19 (38.8%)	
> 60	7 (77.8%)	30 (61.2%)	
Gender			0.079
Female	9 (100.0%)	36 (73.5%)	
Male	0 (00.0%)	13 (26.5%)	
Multiple tumor nodules			0.376
Positive	1 (1.1%)	12 (24.5%)	
Negative	8 (88.9%)	37 (75.5%)	
Tumor size, mm			0.114
≤ 50	0 (00.0%)	11 (22.4%)	
> 50	9 (100.0%)	38 (77.6%)	
Angioinvasion			0.634
Positive	4 (44.4%)	26 (53.1%)	
Negative	5 (55.6%)	23 (46.9%)	
Histologic differentiation			0.786
Well	2 (22.2%)	9 (18.4%)	
Moderate/poor	7 (77.8%)	40 (81.6%)	
Pathologic T stage			0.079
T1/T2	7 (77.8%)	22 (45.8%)	
T3/T4	2 (22.2%)	27 (54.2%)	
Pathologic N stage			0.537
Positive	0 (00.0%)	2 (4.1%)	
Negative	9 (100.0%)	47(95.9%)	
TEMs in TIF			0.132
Positive	8 (88.9%)	31 (63.3%)	
Negative	1 (11.1%)	18 (36.7%)	
Operative variables			
R status			0.691
Positive	1 (11.1%)	8 (16.3%)	
Negative	8 (88.9%)	41 (83.7%)	
Variables of patient outcome			
Local tumor recurrence			0.007
Positive	6 (66.7%)	11 (22.4%)	
Negative	3 (33.3%)	38 (77.6%)	
Overall tumor recurrence			0.072
Positive	6 (66.7%)	17 (34.7%)	
Negative	3 (33.3%)	32 (65.3%)	
Metastases			0.513
Positive	1 (1.1%)	10 (20.4%)	
Negative	8 (88.9%)	39 (79.6%)	
Variable	ANG1 high/TCA	ANG1 low/TCA	*p*
No. of patients	12	46	
Patient- and tumor-related variables			
TEMs in TCA			0.111
Positive	5 (41.7%)	9 (19.6%)	
Negative	7 (58.3%)	37 (80.4%)	
TEMs in TIF			0.182
Positive	10 (83.3%)	29 (63.0%)	
Negative	2 (16.7%)	17 (36.0%)	
Angioinvasion			0.245
Positive	8 (66.7%)	22 (47.8%)	
Negative	4 (33.3%)	24 (52.2%)	
Variables of patient outcome			
Metastases			0.024
Positive	5 (41.7%)	6 (13.0%)	
Negative	7 (58.3%)	40 (87.0%)	

The Fischer test was applied when the number of patients in the subgroups was less than five (*n* < 5) in more than 25% of cases

**Table 3:** Association of histologic tumor necrosis with clinicopathological characteristics of the patients with hepatocellular carcinoma (HCC) as determined by the chi-squared (*χ*^2^) test

Variable	Necrosis^+^	Necrosis	*p*
No. of patients	35	23	
Patient- and tumor-related variables			
Patient age, years			0.707
≤ 60	12 (34.3%)	9 (39.1%)	
> 60	23 (65.7%)	14 (60.9%)	
Gender			0.920
Female	27 (77.1%)	18 (78.3%)	
Male	8 (22.9%)	5 (21.7%)	
Multiple tumor nodules			0.587
Positive	7 (20.0%)	6 (26.1%)	
Negative	28 (80.0%)	17 (73.9%)	
Tumor size, mm			0.001
≤ 50	2 (5.7%)	9 (39.1%)	
> 50	33 (94.3%)	14 (60.9%)	
Angioinvasion			0.120
Positive	21 (60.0%)	9 (39.1%)	
Negative	14 (40.0%)	14 (60.9%)	
Lymphangiosis carcinomatosa			0.304
Positive	12 (34.3%)	5 (21.7%)	
Negative	23 (65.7%)	18 (78.3%)	
Histologic differentiation			0.262
Well	5 (14.3%)	6 (26.1%)	
Moderate/poor	30 (85.7%)	17 (73.9%)	
Pathologic T stage			0.038
T1/T2	14 (40.0%)	15 (68.2%)	
T3/T4	21 (60.0%)	8 (31.8%)	
Pathologic N stage			0.076
Positive	0 (00.0%)	2 (8.7%)	
Negative	35 (100.0%)	21 (91.3%)	
TEMs in TCA			0.026
Positive	12 (34.3%)	2 (8.7%)	
Negative	23 (65.7%)	21 (91.3%)	
TEMs in TIF			0.402
Positive	25 (71.4%)	14 (60.9%)	
Negative	10 (28.6%)	9 (39.1%)	
Angiopoietin-1 in TCA			0.149
Positive	3 (8.6%)	0 (00.0%)	
Negative	32 (91.4%)	23 (100.0%)	
Angiopoietin-1 in TIF			0.245
Positive	28 (80.0%)	21 (91.3%)	
Negative	7 (20.0%)	2 (8.7%)	
Angiopoietin-2 in TCA			0.818
Positive	2 (5.7%)	1 (4.3%)	
Negative	33 (94.3%)	22 (95.7%)	
Angiopoietin-2 in TIF			0.326
Positive	1 (2.9%)	2 (8.7%)	
Negative	34 (97.1%)	21 (91.3%)	
Operative variables			
R status			0.245
Positive	7 (20.0%)	2 (8.7%)	
Negative	28 (80.0%)	21 (91.3%)	
Variables of patient outcome			
Local tumor recurrence			0.662
Positive	11 (31.4%)	6 (26.1%)	
Negative	24 (68.6%)	17 (73.9%)	
Overall tumor recurrence			0.087
Positive	17 (48.6%)	6 (26.1%)	
Negative	18 (51.4%)	17 (73.9%)	
Metastases			0.103
Positive	9 (25.7%)	2 (8.7%)	
Negative	26 (74.3%)	21 (91.3%)	

The Fischer test was applied when the number of patients in the subgroups was less than five (*n* < 5) in more than 25% of cases

**Table 4:** Multivariate analysis of prognostic factors in patients with hepatocellular carcinoma (HCC) conducted in a step-wise manner

Variable	Category	Odds ratio	*p*	Confidence interval
Overall survival				
Distant metastases	Negative (*n* = 47)	0.605	0.602	0.091–4.011
	Positive (*n* = 11)			
Overall tumor recurrence	Negative (*n* = 35)	1.828	0.272	0.623–5.368
	Positive (*n* = 23)			
Angioinvasion	Negative (*n* = 28)	0.286	0.040	0.086–0.945
	Positive (*n* = 30)			
Lymphangiosis carcinomatosa	Negative (*n* = 41)	1.700	0.330	0.585–4.940
	Positive (*n* = 17)			
T status	T1&T2 (*n* = 29)	18.606	0.001	3.397–101.904
	T3&T4	(*n* = 29)		
Angiopoietin-1 in TCA	Negative (*n* = 55)	4.859	0.021	1.274–18.537
	Positive (*n* = 3)			
TEMs in TCA	Negative (*n* = 44)	2.837	0.051	0.997–8.071
	Positive (*n* = 14)			
Recurrence-free survival				
R status	Negative (*n* = 49)	4.346	0.061	0.936–20.174
	Positive (*n* = 9)			
T status	T1&T2 (*n* = 29)	11.213	0.0001	2.997–41.957
	T3&T4	(*n* = 29)		
Angioinvasion	Negative (*n* = 28)	0.458	0.122	0.170–1.234
	Positive (*n* = 30)			
Lymph node involvement	Negative (*n* = 56)	0.311	0.255	0.042–2.322
	Positive (*n* = 56)			
TEMs in TCA	Negative (*n* = 44)	0.391	0.083	0.135–1.132
	Positive (*n* = 14)			
TEMs in TIF	Negative (*n* = 19)	3.677	0.049	1.1007–13.430
	Positive (*n* = 39)			
Angiopoietin-1 in TIF	Negative (*n* = 9)	0.102	0.001	0.028–0.380
	Positive (*n* = 49)			
Angiopoietin-1 in TCA	Negative (*n* = 55)	20.920	0.004	2.620–167.067
	Positive (*n* = 3)			

### Tumor necrosis is associated with an advanced tumor stage and TIE2-expressing monocytes in patients with HCC

Typical images for the occurrence of tumor necrosis in HCC are shown in Fig. 2. The statistical data of the patients is summarized in Table 3. Tumor necrosis was associated with an advanced tumor stage (tumor stage T3/T4; *p* = 0.038). In the necrosis^+^ group, 21/35 (60.0%) patients showed an advanced T stage, whereas in the necrosis^−^ group, only 8/23 (31.8%) patients revealed this feature. Furthermore, tumor necrosis was associated with intensified TEM infiltration in HCC (Fig. 2, Table 3). In the necrosis^−^ group, infiltrating TEMs in TCA were detected in only 2/23 (8.7%) patients. In comparison, in the necrosis^+^ group, 12/35 (34.3%) patients showed tumor-infiltrating TEMs (*p* = 0.026).

### Influence on survival and prognostic significance of angiopoietins, TIE2-expressing monocytes and tumor necrosis in HCC patients

**Fig. 3:**
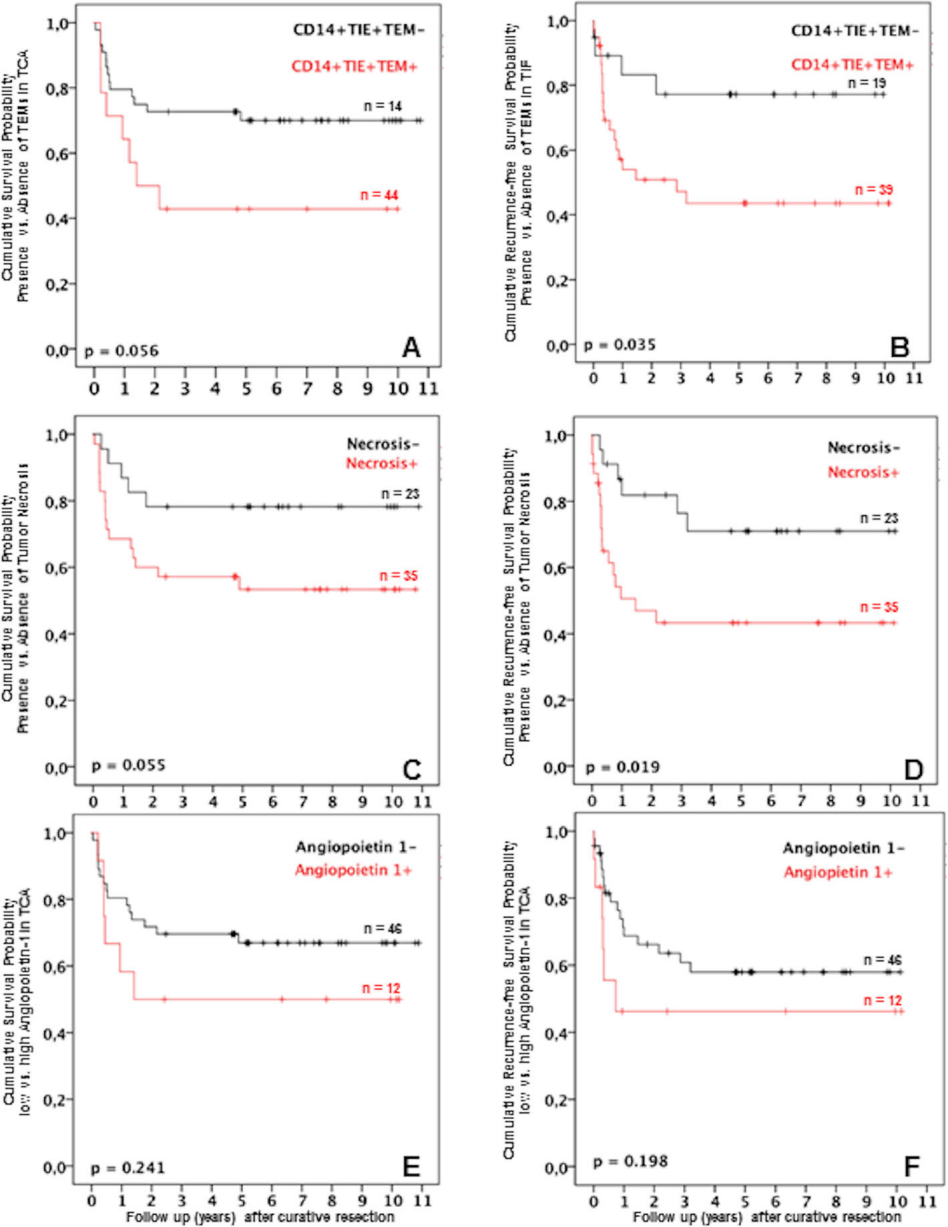
Association of TIE2-expressing monocytes (TEMs), tumor necrosis, and angiopoietins with patient survival using the Kaplan–Meier survival analysis. **a** Overall survival after hepatocellular carcinoma (HCC) surgery in relation to presence or absence of TEMs in the tumor central area (TCA). **b** Recurrence-free survival after HCC surgery in relation to presence or absence of TEMs in the tumor-infiltrating front (TIF). **c** Survival after HCC surgery in relation to presence or absence of histologic tumor necrosis. **d** Recurrence-free survival after HCC surgery in relation to presence or absence of histologic tumor necrosis. **e** Overall survival after HCC surgery in relation to low or high angiopoietin expression in the TCA. **f** Recurrence-free survival after HCC surgery in relation to low or high angiopoietin expression in the TCA

In the present study, TEMs and histologic tumor necrosis was associated with patients’ overall survival and recurrence-free survival after liver resection for HCC. The Kaplan–Meier survival curves are shown in Fig. 3. The statistical data of all patients is summarized in Tables 1, 2, 3, and 4. After liver resection for HCC, survival was decreased in patients with TEMs in TCA (*p* = 0.056) when compared to patients without these cells in the TCA. The overall 1-, 3-, and 5-year survival rates were 63.3%, 42.8%, and 42.8%, respectively, in the TEM^+^ group as compared to 79.6%, 77.1%, and 75.0%, respectively, in the TEM^−^ group (Fig. 3a). Furthermore, the presence of TEMs in the TIF was associated with a reduced recurrence-free survival. The 1-, 3-, and 5-year recurrence-free survival of patients with TEMs were lower (52.0%, 43.3%, and 42.2%, respectively) as compared to patients without TEMs (83.1%, 77.9%, and 77.9%, respectively) (*p* = 0.035) (Fig. 3b). Patients in the necrosis^−^ group had improved overall survival and recurrence-free survival when compared with the necrosis^+^ group (Fig. 3c, d; *p* = 0.055 and *p* = 0.019, respectively). In addition, we analyzed whether angiopoietins, infiltrating TEMs, and histologic tumor necrosis could predict patient outcomes after surgery for HCC. Using a step-wise multivariate analysis, in addition to other established clinicopathological parameters, angiopoietin-1 and TEMs were identified as independent prognostic factors for both overall survival and recurrence-free survival (Table 4). In our work, the presence of liver steatosis was not associated with patient outcomes following resection. The Additional file 3 provides a synopsis concerning the results with liver steatosis.

## Discussion

In our work, the tissue densities of angiopoietins and angiopoietin receptor-bearing monocytes/macrophages as well as the occurrence of histologic tumor necrosis in specimens of HCC patients were associated with clinicopathologic characteristics and patient survival. The main findings were as follows: (1) angiopoietins and infiltrating TEMs were associated with metastatic and recurrent disease; (2) the presence of tumor necrosis was associated with an advanced tumor stage and associated with TEM frequency; and (3) necrosis and TEMs exerted a pernicious influence on patient survival.

In this study, we demonstrated that high angiopoietin expression and the presence of TEMs was associated with recurrent and metastatic HCC. These results are in accordance with previously published results on HCC and other hepatobiliary tumors, which delineate the negative impact of infiltrating monocytes/macrophages on patient survival and outcome [34–37]. Angiopoietins represent a potential prognostic biomarker of therapeutic responsiveness to anticancer treatments, including immunotherapy using immunomodulatory agents [38]. The activation of the angiopoietin-TIE2 axis could be a potent tumor escape mechanism from anti-angiogenic treatment. Novel data implicates that, in HCC, angiopoietins are co-expressed with VEGF and transiently decrease during the window of normalization and return to baseline levels after anti-VEGF therapy [38]. Tumor-derived VEGF can increase the tumor expression of angiopoietins and promote metastases, suggesting that interactions between these angiogenic pathways promote tumor progression [39]. Furthermore, several preclinical studies have shown that monocytes/macrophages, and especially angiopoietin receptors-bearing TEMs, contribute to tumor neoangiogenesis in mouse tumor models [40]. Therefore, these monocytes/macrophages may exert potent abilities to enhance the extent of tumor neoangiogenesis and progression to increased malignancy in patients with HCC. These findings suggest that angiopoietin signaling may foster the invasion of monocytes/macrophages, resulting in deleterious effects in HCC; however, further studies are needed to elucidate the possible functional mechanisms and help conceptualize novel checkpoint inhibitor targets for cancer immunotherapy.

HCC is a highly vascularized tumor and the recurrent disease remains a major clinical obstacle and defines the outcome of the patients receiving curative therapy. In the current work, we provide data that could be used to identify subgroups of patients with advantageous or deleterious HCC characteristics, respectively. Such a personalized approach may have a useful clinical implication in order to improve the individualized risk stratification after surgery and curative treatments. This translates ultimately into more intensified and purposeful aftercare of patients with HCC at high risk for adverse outcomes. De Palma et al. were the first to report on the significance of TEMs in regard to tumor neoangiogenesis [18]. In the clinical setting, Matsubara et al. reported first on the importance of TEMs in human blood and associated their presence with tumor neoangiogenesis, recurrence, and patient survival rates [41]. Consequently, our group reported on the prognostic value of TEMs and histologic tumor necrosis in other hepatobiliary tumors [19–21, 25, 26]. Here, we showed that tumor necrosis and the presence of TEMs and angiopoietins were associated with an advanced HCC. In the current work, tumor necrosis was also associated with TEM density in HCC. These characteristics of the tumor microenvironment also affected overall survival and recurrence-free survival of the patients. Thus, we propose a coherent construct comprising tumor necrosis, neoangiogenesis, and associated TEMs as an attractive diagnostic modality prognosticating the outcome of HCC patients following radical surgery.

The clinical translation of novel immunologic aspects of the tumor biology represents an urgent unmet need. In addition, a major clinical problem is the lack of adjuvant treatment strategies in patients with advanced HCC [34]. Novel experimental techniques demonstrated high efficacy in visualization of important localized sites of the tumor microenvironment and related tumor necrosis. Moreover, these approaches offer the possibility for nanoparticles uptake and consequent clinical imaging of monocytes/macrophages in the vicinity of the tumor, as well. Aghighi et al. established a novel MRI-based modality for selective visualization of the tumor central area and the infiltrating margin that is adjacent to normal non-cancerous tissues [42]. In this setting, the utilization of ultra-small superparamagnetic iron oxide nanoparticles in ferumoxytol-enhanced MRI provides an opportunity to better characterize the extra- and intracellular compartments of solid tumors, corresponding TAMs, and formation of necrosis in the tumor microenvironment, which is a novel diagnostic tool with a high sensitivity and potential for immediate clinical translation [42, 43]. Moreover, a novel tumor necrosis therapy (TNT), which provides a new and promising therapeutic anticancer modality, has been recently described, as well [44]. Through radiolabeled necrosis avid compounds (i.e., small molecules with a high affinity for and long-lasting retention in necrotic tissues), tumor necrosis can be a carrier of therapeutic radionuclides [45–47].

## Conclusions

In conclusion, formation of histologic tumor necrosis is associated with the intratumoral density of invading angiogenic TEMs of HCC patients. Furthermore, the tumor necrosis–TEM–angiopoietin axis was associated with disease recurrence, advanced tumor stage, and reduced survival after radical surgery in these patients. Thus, our data may indicate the tumor necrosis–TEM–angiopoietin axis is a novel checkpoint target in tumor immunotherapy and, at the same time, provides potent anti-angiogenesis modalities in advanced HCC. However, some limitations of the current study should be taken into account. The small number of cases in the different subgroups remains the main drawback. On the other hand, our work incorporated no functional studies and remains a descriptive study. Therefore, further research encompassing larger patient populations with focus on functional assays and mechanistic links is urgently needed. This will deliver a deeper biological insight into possible mechanistic molecular pathways might help develop novel immunologic checkpoint inhibitor targets for hepatic malignancies.

## Supplementary information

**Additional file 1: Figure S1.** Flowchart describing the patient selection process for our study.

**Additional file 2: Figure S2.** Negative control used in the immunohistology, showing also representative sites of the tumor central area (TCA) and infiltrating front (TIF). The dashed line marks the representative boundary between TCA and TIF. The TIF was defined as the microscopic area localized in direct proximity, i.e., next to the adjacent normal liver tissue. The TCA was defined as the tumor tissue that is surrounded by the infiltrating front and has no contact with normal hepatocytes. Scale bar 50 μm.

**Additional file 3: Table S1.** Antibodies and reagents used for immunohistology. **Table S2.** Association of the presence of liver steatosis with clinicopathological characteristics of patients with hepatocellular carcinoma as determined by the chi-squared (*χ*^2^) test. The Fischer test was applied when the number of patients in the subgroups was less than five (*n* < 5) in more than 25% of cases.

## Data Availability

The datasets used and/or analyzed during the current study are available from the corresponding author on reasonable request.

## Abbreviations

AFP: Alpha-fetoprotein
Ang: Angiopoietin
CCA: Cholangiocarcinoma
CT: Computed tomography
HCC: Hepatocellular carcinoma
MRI: Magnetic resonance imaging
NASH: Non-alcoholic steatohepatitis
PDAC: Periductal adenocarcinoma of the pancreas
TAMs: Tumor-associated macrophages
TCA: Tumor central area
TEMs: TIE2-expressing monocytes
TIF: Tumor-infiltrating front
TNT: Tumor necrosis therapy
VEGF: Vascular endothelial growth factor
