# Supplementary figures and images for: Angiogenic inflammation and formation of necrosis in the tumor microenvironment influence patient survival after radical surgery for de novo hepatocellular carcinoma in non-cirrhosis
Source: World J Surg Oncol. 2019 Dec 12;17:217. doi: 10.1186/s12957-019-1756-8 (PMC6909650; doi:10.1186/s12957-019-1756-8)

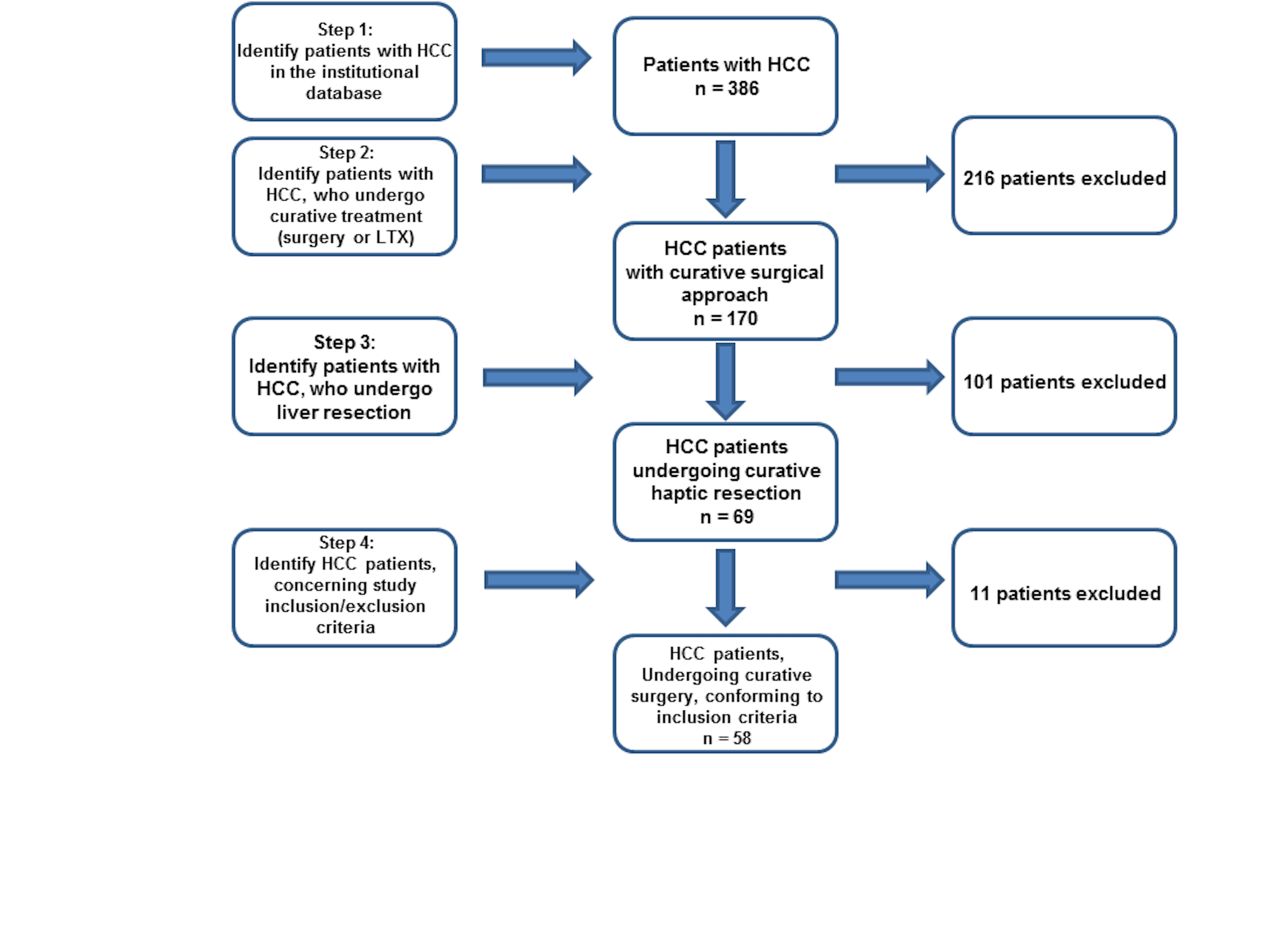

Supplement: Supplementary file 1 — Additional file 1: Figure S1. Flowchart describing the patient selection process for our study. [file 12957_2019_1756_MOESM1_ESM.tif]

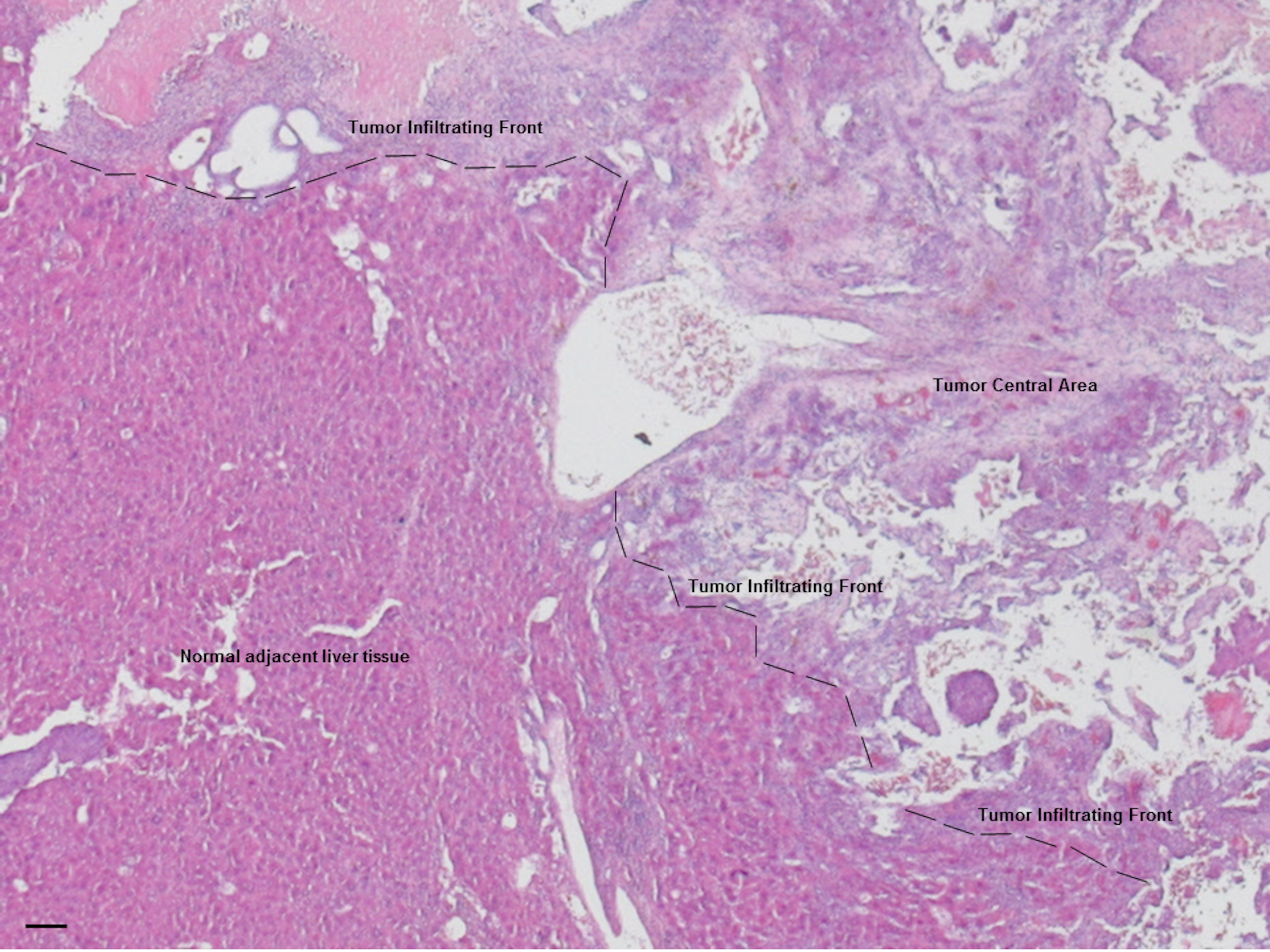

Supplement: Supplementary file 2 — Additional file 2: Figure S2. Negative control used in the immunohistology, showing also representative sites of the tumor central area (TCA) and infiltrating front (TIF). The dashed line marks the representative boundary between TCA and TIF. The TIF was defined as the microscopic area localized in direct proximity, i.e., next to the adjacent normal liver tissue. The TCA was defined as the tumor tissue that is surrounded by the infiltrating front and has no contact with normal hepatocytes. Scale bar 50 μm. [file 12957_2019_1756_MOESM2_ESM.tif]
