# Supplementary material for: Angiogenic inflammation and formation of necrosis in the tumor microenvironment influence patient survival after radical surgery for de novo hepatocellular carcinoma in non-cirrhosis
Source: World J Surg Oncol. 2019 Dec 12;17:217. doi: 10.1186/s12957-019-1756-8 (PMC6909650; doi:10.1186/s12957-019-1756-8)
Supplement: Supplementary file 3 — Additional file 3: Table S1. Antibodies and reagents used for immunohistology. Table S2. Association of the presence of liver steatosis with clinicopathological characteristics of patients with hepatocellular carcinoma as determined by the chi-squared (χ2) test. The Fischer test was applied when the number of patients in the subgroups was less than five (n < 5) in more than 25% of cases. [file 12957_2019_1756_MOESM3_ESM.docx]

**Additional file**

***Study design, patients and tumor samples***

The study period began on April 10, 2002 and ended on April 10, 2015. Patients (n = 58) with histopathologically confirmed hepatocellular carcinoma (HCC), who received a major hepatectomy in a curative intent, were included in our study. In the current work, the following inclusion criteria concerning patients with curative surgery were applied:

1. A meaningful oncologic approach as assessed by the institutional interdisciplinary tumor board
2. Technically resectable tumor mass
3. Absence of metastatic spread to distant organs

No pediatric patients were included in the study, and none of the patients received antiangiogenic treatment modalities (i.e., sorafenib) prior to surgery. Patients with HCC who died within 60 days after the surgery were excluded from the study design. Supplementary Figure 1 depicts a flowchart describing the patient selection process for our study.

Concerning studies in tumor immunology, one should bear in mind whether the tumor biology of the studied patients is naive/normal or already somehow altered due to neoadjuvant treatment strategies (i.e., TACE or RFA). In our hypothesis, unaltered tumor biology is considered to deliver insights concerning tumor immunology that would reflect the normal nature and functionality of the tumor microenvironment of HCC. Therefore, we designed the concept of our study comprising patients with no pretreatment (i.e., patients with normal tumor biology).

***Immunohistology for angiopoietins*.**

The supplementary table 1 summarizes the reagents used. Formalin-fixed and paraffin-embedded tumor sections (5 μm thick) were dewaxed and rehydrated. The anti-retrieval was performed with a different solution (Table 1) in a streamer. Additional endogenous peroxidase activity was inhibited with 3% H_2_O, and endogenous biotin was suppressed by sequential incubation in 0.1% avidin and 0.01% biotin (Dako, Glostrup, Denmark). The pAb goat Ang-1 (N-18) and pAb goat Ang-2 (N-18) (both Santa Cruz Biotechno­logy, California, USA) were incubated for 30 min at room temperature. The visualization of the antibody reaction was performed with LSAB 2 System-HRP and DAB+ liquid substrate chromogen system (Dako, Glostrup, Denmark). Sections were counterstained with hematoxylin. Specificity controls were performed without the primary antibodies.

***Immunohistology for angiogenic monocytes/macrophages*.**

In the current work, to distinguish between monocytes/macrophages (double positive for CD14 and TIE2) and microvessels (positive only for TIE2), we conducted immunostaining for simultaneous detection of CD14 and TIE2. Therefore, the corresponding monocytes (i.e., TEMs) are visualized as cells that are double positive for the above biomarkers. This positivity in the immunohistology is seen as simultaneous red and brown staining. We carefully selected the corresponding color reaction (red and brown) of the secondary antibody to ensure that a good contrast for accurate histological evaluation was guaranteed. On the other hand, microvessels (i.e., endothelial cells) were positive only for TIE2 and were, thus, visualized as brown reactivity only.

Immunohistological double staining was performed for TEMs. Tissue sections were dewaxed and rehydrated. Antigen retrieval was performed by heating the slides in target retrieval solution pH 6.1 (Dako, Glostrup, Denmark) in a pressure cooker and subsequent cooling on ice for 30 min each. Endogenous peroxidase activity was inhibited with 3% H_2_O_2_ in methanol for 20 min at 4°C. Nonspecific binding sites were blocked with 2.5% normal horse serum for 20 min. Tissue sections were incubated with polyclonal goat antibody anti-human Tie-2 (1:80, R&D Systems Minneapolis, USA) overnight at 4°C. ImmPRESS REAGENT-Anti-Goat Ig, peroxidase and ImmPACT DAB Peroxidase Substrate (both Vector Laboratories, Burlingame, Canada) were applied, as per description, for the visualization of the antibody reaction. A renewed blockade of the nonspecific binding sites was performed with 2.5% normal horse serum. In a next step, tissue sections were incubated with the polyclonal rabbit antibody anti-human CD14 (1:400, Sigma-Aldrich, Munich, Germany) overnight at 4°C. For the visualization of the CD14 antibody reaction, the ImmPRESS REAGENT-Anti-Rabbit Ig, alkaline phosphatase and Vector Red Alkaline Phosphatase Substrate Kit I (both Vector Laboratories, Burlingame, Canada) with levamisole (Vector Laboratories, Burlingame, Canada) were applied. Sections were counterstained with hematoxylin. Specificity controls were performed without the primary antibodies.

***Histological evaluation of tumor central area and infiltrating front***

The tumor-infiltrating front was defined as the microscopic area localized in direct proximity (i.e., next to the adjacent normal liver tissue). The tumor central area was defined as the tumor tissue that is surrounded by the infiltration front and has no contact with normal hepatocytes (Supplementary Figure 2).

***Study of HCC Recurrence***

HCC recurrence is a central focus in our study. Tumor recurrence with lethal complication proved to be the most common cause of death in the long term in the patients with HCC. In 21/58 (36.2%) cases death occurred in the further course after HCC resection. Of the deceased patients, 11/21 (52.4%) had HCC recurrence with fatal complications. Local HCC recurrence with concomitant metastatic spread was observed in 3/55 (5.2%) patients. Local recurrence without concomitant metastasis was seen in 14/58 (24.1%) patients. Altogether, metastatic spread to distant organs was detected in 9/58 (15.5%) patients.

***Analysis and quantification of liver steatosis***

Recently, we reported on the demographics, clinicopathologic characteristics and clinical significance of tumor-associated lymphocytes and alternatively activated monocytes/macrophages in patients with de novo HCC (1). We conducted the current work to look deeper into the backgrounds and possible etiology of the liver disease in cases with de novo HCC. Each fifth HCC patient worldwide has no history of underlying liver disease; however, accumulating data demonstrates that, in a considerable number of these de novo HCC patients, liver steatosis is present and is mechanistically linked to hepatocarcinogenesis (2, 3). Thus, we evaluated the frequency of liver steatosis in the patient population of this study. Accumulating studies on the importance of liver steatosis as a risk factor for liver disease report coherently on the quantification of steatosis in “absence or presence.” In addition, the presence of steatosis is further quantified into mild steatosis (>5% and <30%), moderate steatosis (30–60%) and severe steatosis (>60%) (4-8). Therefore, in our study, the pathological evaluation of the degree of liver steatosis was carried out with the above published results and classifications. In the current work, severe steatosis was observed in 6/58 (6.9%) patients with HCC. Another 11/58 (19%) patients had moderate steatosis. Mild steatosis was detected in the tumor samples of 19/58 (30.0%) patients.

***Liver steatosis associates inversely with angiopoietin density in HCC***

The supplementary table 2 summarizes the results concerning liver steatosis. The density level of angiopoietin-1 in the TCA was associated inversely with the presence of liver steatosis. In the ANG1^low^ group, 32/36 (88.9%) patients had liver steatosis. On the other hand, there were 14/22 (63.6%) cases in the ANG1^high^ group (p = 0.021). No significant association of steatosis with overall survival or recurrence-free survival was detected. Moreover, no statistical associations with further clinicopathological variables were seen. In a next step, the subgroup analysis of patients with the absence of, mild, moderate and severe steatosis was conducted. Again, no statistically significant p values were observed (data not shown).

The results concerning liver steatosis presented in the current work are in conformity with most of the data published in the literature demonstrating that steatosis-associated HCC delivered similar overall survival and recurrence-free survival rates after resection as compared to HCC outcome after resection for other etiologies (9-11). On the other hand, several studies revealed that steatosis-associated HCC was associated with a deteriorated outcome after resection. However, the analyzed subgroup of patients with steatosis comprised significantly older patients with increased rates of cardiovascular burden, metastatic spread, and larger tumor size (12, 13).

***TIE2-expressing monocytes are associated with local tumor recurrence***

Our group previously demonstrated the importance of TEMs in bile cancer and adenocarcinoma of the pancreas (14, 15). In our work, TEMs are preferentially located in regions of tumor necrosis and neovascularization (Figure 1A–1D). In addition to overall tumor recurrence, these immune cells were also associated with local HCC recurrence following hepatic resection. When considering the TIF, in the TEM^-^ group, only 1/19 (5.3%) patients had a local tumor recurrence, whereas in the TEM^+^ group, 16/39 (41.0%) patients had this phenomenon (p = 0.005). In addition, the presence of TEMs in the TIF showed a trend towards more frequent angioinvasion in patients with HCC (p = 0.113) (table 1).

***Angiopoietins are associated with metastatic disease in patients with HCC***

Low frequency of angiopoietin-1 in the TCA was associated with a reduced incidence of metastatic disease (p = 0.024). In the ANG1^low^ group, only 6/46 (13.0%) patients had metastases to distant organs, whereas in the ANG1^high^ group, 5/12 (41.7%) patients had metastases to distant organs. In addition, angiopoietin tumor density also showed a trend towards more frequent angioinvasion, tumor recurrence and larger tumor size in patients with HCC, without reaching statistical significance (Table 2, main text). Trends towards reduced overall survival and recurrence-free survival after resection were noted in those patients with high angiopoietin-1 frequency in the TCA; however, this observation did not reach statistical significance (Figures 3E and 3F; p = 0.241 and p = 0.198, respectively).

***Tumor necrosis is associated with tumor size in patients with HCC***

We have previously shown that the formation of tumor necrosis was associated with monocyte/macrophage density and was also associated with patient survival and outcome after radical surgery for hilar and intrahepatic cholangiocarcinoma (16, 17). In the current work, the tumor samples of 23 patients (39.7%) displayed no tumor necrosis (Necrosis^-^ group), while necrosis was evident (Necrosis^+^ group) in 35 cases (60.3%). Formation of histologic tumor necrosis was also associated with a larger tumor size. In the Necrosis^+^ group, 33/35 (94.3%) patients had larger tumor size (largest tumor diameter > 50 mm); in the Necrosis^-^ group, this feature was seen only in 14/23 (60.9%) patients (p = 0.001). In addition, tumor necrosis revealed trends towards enhanced angioinvasion, metastases, tumor recurrence and intensified angiopoietin tumor density in patients with HCC (Table 3).

In cases of rapidly growing tumors, the formation of necrosis can be certainly due to the relative hypoperfusion and the tumor size. Indeed, in this work, we showed that necrosis and tumor size are associated. However, accumulating scientific data indicates that the occurrence of histologic tumor necrosis is functionally mediated by infiltrating monocytes/macrophages (18). Therefore, in the current work, we explored the possibility of tumor necrosis being facilitated by immunological components of the tumor microenvironment (i.e., invading monocytes/macrophages). In addition, we were able to show that these cells were associated with the formation of tumor necrosis in the tumor specimen, but not with tumor size.

**REFERENCES (SUPPLEMENTARY MATERIAL)**

1. Atanasov G, Dino K, Schierle K, Dietel C, Aust G, Pratschke J, et al. Immunologic cellular characteristics of the tumour microenvironment of hepatocellular carcinoma drive patient outcomes. World J Surg Oncol. 2019;17:97.
2. Dhamija E, Paul SB, Kedia S. Non-alcoholic fatty liver disease associated with hepatocellular carcinoma: an increasing concern. Indian J Med Res. 2019;149:9-17.
3. Fujiwara N, Friedman SL, Goossens N, Hoshida Y. Risk factors and prevention of hepatocellular carcinoma in the era of precision medicine. J Hepatol. 2018;68:526-49.
4. de Meijer VE, Kalish BT, Puder M, Ijzermans JN. Systematic review and meta-analysis of steatosis as a risk factor in major hepatic resection*.* Br J Surg. 2010;97:1331-9.
5. Gomez D, Malik HZ, Bonney GK, Wong V, Toogood GJ, Lodge JP, et al. Steatosis predicts postoperative morbidity following hepatic resection for colorectal metastasis. Br J Surg. 2007;94:1395-402.
6. McCormack L, Petrowsky H, Jochum W, Furrer K, Clavien PA. Hepatic steatosis is a risk factor for postoperative complications after major hepatectomy: a matched case-control study. Ann Surg. 2007;245:923-30.
7. Kooby DA, Fong Y, Suriawinata A, Gonen M, Allen PJ, Klimstra DS, et al. Impact of steatosis on perioperative outcome following hepatic resection. J Gastrointest Surg. 2003;7:1034-44.
8. Behrns KE, Tsiotos GG, DeSouza NF, Krishna MK, Ludwig J, Nagorney DM. Hepatic steatosis as a potential risk factor for major hepatic resection. J Gastrointest Surg. 1998;2:292-8.
9. Pais R, Fartoux L, Goumard C, Scatton O, Wendum D, Rosmorduc O, et al. Temporal trends, clinical patterns and outcomes of NAFLD-related HCC in patients undergoing liver resection over a 20-year period. Aliment Pharmacol Ther. 2017;46:856-63.
10. Yang T, Hu LY, Li ZL, Liu K, Wu H, Xing H, et al. Liver resection for hepatocellular carcinoma in non-alcoholic fatty liver disease: a multicenter propensity matching analysis with HBV-HCC. J Gastrointest Surg. 2019; doi: 10.1007/s11605-018-04071-2.
11. Viganò L, Conci S, Cescon M, Fava C, Capelli P, D'Errico A, et al. Liver resection for hepatocellular carcinoma in patients with metabolic syndrome: a multicenter matched analysis with HCV-related HCC. J Hepatol. 2015;63:93-101.
12. Wong CR, Njei B, Nguyen MH, Nguyen A, Lim JK. Survival after treatment with curative intent for hepatocellular carcinoma among patients with vs without non-alcoholic fatty liver disease. Aliment Pharmacol Ther. 2017;46:1061-9.
13. Koh YX, Tan HJ, Liew YX, Syn N, Teo JY, Lee SY, et al. Liver resection for nonalcoholic fatty liver disease-associated hepatocellular carcinoma. J Am Coll Surg. 2019. pii: S1072-7515(19)30448-X.
14. Atanasov G, Pötner C, Aust G, Schierle K, Dietel C, Benzing C, et al. TIE2-expressing monocytes and M2-polarized macrophages impact survival and correlate with angiogenesis in adenocarcinoma of the pancreas. Oncotarget*.* 2018;9:29715-26.
15. Atanasov G, Dietel C, Feldbrügge L, Benzing C, Krenzien F, Brandl A, et al. Angiogenic miRNAs, the angiopoietin axis and related TIE2-expressing monocytes affect outcomes in cholangiocarcinoma. Oncotarget*.* 2018;9:29921-33.
16. Atanasov G, Schierle K, Hau HM, Dietel C, Krenzien F, Brandl A, et al. Prognostic significance of tumor necrosis in hilar cholangiocarcinoma. Ann Surg Oncol*.* 2017;24:518-25.
17. Atanasov G, Dietel C, Feldbrügge L, Benzing C, Krenzien F, Brandl A, et al. Tumor necrosis and infiltrating macrophages predict survival after curative resection for cholangiocarcinoma. *OncoImmunology.* 2017, Jun 28;6(8):e1331806
18. Richards CH, Mohammed Z, Qayyum T, Horgan PG, McMillin DC. The prognostic value of histological tumor necrosis in solid organ malignant disease: a systematic review. Future Oncol*.* 2011;7:1223–35.

**Figure S1** Flowchart describing the patient selection process for our study.

**Figure S2** Negative control used in the immunohistology, showing also representative sites of the tumor central area (TCA) and infiltrating front (TIF). The dashed line marks the representative boundary between TCA and TIF. The TIF was defined as the microscopic area localized in direct proximity, i.e., next to the adjacent normal liver tissue. The TCA was defined as the tumor tissue that is surrounded by the infiltrating front and has no contact with normal hepatocytes. Scale bar 50 µm.

**Table S1** Antibodies and reagents used for immunohistology.

| **Primary Antibody** | **Species** | **Dilution** | **Antigen Retrieval Solution** | **Secondary Antibody System** | **Species** |
| --- | --- | --- | --- | --- | --- |
| pAb goat Ang-1 (N-18) | Human | 1:50 | 10 mM Citrate Buffer (pH 5,5) | Universal  LSAB+ system-HRP | Rabbit, mouse, goat |
| pAb goat Ang-2 (N-18) | Human | 1:50 | 10 mM Citrate Buffer (pH 5,5) | Universal  LSAB+ system-HRP | Rabbit, mouse, goat |
| pAb goat  Human Tie-2 | Human | 1:80 | Target Retrieval Solution (pH 6,1) | ImmPress Reagent  Anti-Goat Ig; Peroxidase | Goat |
| pAb rabbit  Anti-CD14 | Human | 1:400 | Target Retrieval Solution (pH 6,1) | ImmPress  Reagent Anti-Rabbit Ig; Alkaline Phosphatase | Rabbit |

**Table S2** Association of the presence of liver steatosis with clinicopathological characteristics of patients with hepatocellular carcinoma as determined by the chi-squared (*χ*^2^) test. The Fischer test was applied when the number of patients in the subgroups was less than five (n < 5) in more than 25% of cases.

**Variable Absence Presence p**

No. of patients 22 36

**Patient- and tumor-related variables**

Patient age, years 0.587

≤ 60 7 (31.8%) 14 (38.9%)

> 60 15 (68.2%) 22 (61.1%)

Gender 0.103

Female 20 (90.9%) 25 (69.4%)

Male 2 (9.1%) 11 (30.6%)

Multiple tumor nodules 0.210

Positive 3 (13.6%) 10 (27.8%)

Negative 19 (86.4%) 26 (72.2%)

Tumor size, mm 0.418

≤ 50 3 (13.6%) 8 (22.2%)

> 50 19 (86.4%) 28 (77.8%)

Angioinvasion 0.737

Positive 12 (54.5%) 18 (50.0%)

Negative 10 (45.5%) 18 (50.0%)

Lymphangiosis carcinomatosa 0.743 Positive 7 (31.8%) 10 (27.8%)

Negative 15 (68.2%) 26 (72.2%)

Histologic differentiation 0.418

Well 3 (13.6%) 8 (22.2%)

Moderate/poor 19 (86.4%) 28 (77.8%)

Pathologic T stage 0.233

T1/T2 9 (40.9%) 20 (57.1%)

T3/T4 13 (59.1%) 15 (42.9%)

Pathologic N stage 1.000

Positive 1 (4.5%) 1 (2.8%)

Negative 21 (95.5%) 35 (97.2%)

**Operative variables**

R status 0.757

Positive 19 (86.4%) 30 (83.3%)

Negative 3 (13.6%) 6 (16.7%)

**Variables of patient outcome**

Local tumor recurrence 0.083

Positive 7 (31.8%) 4 (11.1%)

Negative 15 (68.2%) 32 (88.9%)

Overall tumor recurrence 0.480

Positive 10 (45.5%) 13 (36.1%)

Negative 12 (54.5%) 23 (63.9%)

Metastases 0.667

Positive 8 (20.5%) 3 (15.8%)

Negative 31 (79.5%) 16 (84.2%)

**Immunologic characteristics**

TEMs/TCA 0.089

Positive 14 (63.6%) 30 (83.3%)

Negative 8 (36.4%) 6 (16.7%)

TEMs/TIF 0.486

Positive 6 (27.3%) 13 (36.1%)

Negative 16 (72.7%) 23 (63.9%)

ANG1/TCA 0.021

Positive 8 (36.4%) 4 (11.1%)

Negative 14 (63.6%) 32 (88.9%)

ANG1/TIF 0.757

Positive 3 (12.6%) 6 (16.7%)

Negative 19 (86.4%) 30 (83.3%)

ANG2/TCA 0.387

Positive 17 (77.3%) 31 (86.1%)

Negative 5 (22.7%) 5 (13.9%)

ANG2/TIF 0.964

Positive 17 (77.3%) 28 (77.8%)

Negative 5 (22.7%) 8 (22.2%)

Tumor necrosis 0.132

Positive 16 (72.7%) 19 (52.8%)

Negative 6 (27.3%) 17 (47.2%)
